# ACTIVITIES OF DAILY LIVING DIFFICULTIES AND TOILETING AMONG OLDER GHANAIANS: AN APPLICATION OF WHO-ICF FRAMEWORK

**DOI:** 10.1093/geroni/igz038.1916

**Published:** 2019-11-08

**Authors:** Kofi Awuviry-Newton, Meredith Tavener, Kylie Wales, Paul Kowal, Julie Byles

**Affiliations:** 1 Priority Research Centre for Generational Health and Ageing, Faculty of Health and Medicine, The University of Newcastle, Newcastle, Australia, Australia; 2 Priority Research Centre for Generational Health and Ageing, Faculty of Health and Medicine, The University of Newcastle, Newcastle, NSW, Australia; 3 School of Health Sciences, The University of Newcastle, Newcastle, NSW, Australia; 4 The University of Newcastle, School of Medicine and Public Health, Faculty of Health and Medicine, Australia; 5 Priority Research Center for generational Health and Ageing, Newcastle, NSW, Australia

## Abstract

The aim of the study was to analyze the prevalence of activities of daily living (ADL) difficulties among older Ghanaians and specifically how one ADL, toileting difficulty, predicts care and supports needs using the World Health Organization International Classification of Disability and Health framework (WHO-ICF). Toileting difficulty requiring upper extremity strength is among ADLs that can lead to functional loss of independence among older people globally. A sample of n=5,096 adults aged 50 years and older from the WHO Study on global AGEing and adult health (SAGE) Ghana Wave 1 was used to analyze difficulties with ADLs and toileting. Level of difficulty was assessed against 22 other functioning items from the interview. Out of the 22 functioning items, climbing one flight of stairs without resting was the most difficult activity to be completed by older Ghanaians, and difficulty eating being the least endorsed item. Toileting was ranked the 16th in terms of reported difficulty and was related to other ADLs. Logistics multivariate regression was used to analyze data. Including significant variables from the univariate analysis in parsimonious model based on WHO-ICF framework, age, self-report health, memory, bodily pain, short distance vision, stroke, neighborhood trust, toilet facility type, and religious meeting attendance, were significantly independently associated with toileting difficulty. Gender was significant at the univariate level but became insignificant after adjusting for body function and structural variables. Toileting difficulty was associated with factors across different components in the WHO-ICF making the WHO-ICF an appropriate tool for understanding health and disability.

